# An analysis of failure rates for treatment options for large to massive irreparable rotator cuff tears: a systematic review

**DOI:** 10.1016/j.jsea.2026.100019

**Published:** 2026-04-13

**Authors:** Sean P. Cooke, Jason L. Koh, Farid Amirouche

**Affiliations:** a Department of Orthopaedic Surgery, University of Illinois at Chicago, Chicago, Illinois, USA; b Department of Orthopaedic Surgery, Northshore University Health System, An Affiliate of the University of Chicago Pritzker School of Medicine, Skokie, Illinois, USA

**Keywords:** Irreparable rotator cuff tears, Massive size, Failure rates, Rotator cuff repair, Reverse total shoulder arthroplasty, Superior capsule reconstruction

## Abstract

**Background:**

A variety of treatment methods for irreparable rotator cuff tears (RCTs) have been proposed, yet there is no conclusive evidence showing any to be superior. This review aims to use failure rates to compare different treatment methods for irreparable RCTs with the goal of identifying which methods are superior.

**Methods:**

The studies in this review included patients with large to massive irreparable RCTs and reported failure rates. Seven treatment categories were identified: arthroscopic débridement, subacromial balloon spacer, interposition graft repair, partial repair, reverse total shoulder arthroplasty, superior capsule reconstruction, and tendon transfers. The failure rates for the groups were compiled and compared, as this was the only common outcome measurement between all studies.

**Results:**

One hundred thirty-six studies consisting of 4,963 shoulders were analyzed. Failure rate, defined as retears, reoperations, or clinical failures as reported, was calculated for each treatment method. The subacromial balloon spacer was found to have the highest failure rate (*P*< .001) when compared to the other surgical treatments. The partial repair group (*P*≤ .001) and the interposition graft group (*P*≤ .012) were in the second highest tier. The arthroscopic débridement, reverse total shoulder arthroplasty, superior capsule reconstruction, and tendon transfer groups were in the superior group with similarly low failure rates (*P*≥ .100).

**Discussion:**

While this review did not identify a single best treatment for irreparable RCTs, it did provide valuable insights into the reliability of different treatments. The results were able to develop a hierarchy based on failure rate, which could help guide treatment for irreparable RCTs.

Rotator cuff tears (RCTs) are very prevalent orthopedic injuries in an aging population that become more common and more often symptomatic with age.^121^ While the gold standard of care is to repair RCTs, there is a subset of RCTs that are classified as irreparable. This determination is made by several different factors and classifications, including the size of the tear (Cofield and Bateman), the presence of fatty infiltration in the rotator cuff muscles (Goutallier and Thomazeau), tendon retraction (Patte), failure of a previous rotator cuff repair, etc.^6^^,^^18^^,^^40^^,^^98^^,^^122^ Irreparable RCTs pose a challenging problem, and there has been a recent focus on the development of novel treatments for irreparable RCTs due to their severely hindering nature to the patients. A paucity of literature has been published comparing different treatment modalities and reviewing the individual treatment methods for irreparable RCTs. However, there is still much discussion on what the best method of treatment is and what the most successful course of treatment should be for a patient suffering from an irreparable RCT.

An initial trial of physical therapy is usually attempted first in the treatment of an irreparable RCT but does not show consistent improvement in function and symptoms, as shown by Kovacevic et al.^60^ The next step is surgical treatment. The determination of which surgical treatment method is based on age, activity level, tear characteristics and classifications, arthritis (Hamada), and the patient's expectations and preferences.^46^ The goal of surgical treatment is to restore function and relieve pain through a variety of methods such as bridging the gap created by the tear using grafts, or partially repairing the tear to provide some return of function, transferring another muscle to reinforce the stability and strength of the shoulder, or using an implant device to reorganize the mechanics of the shoulder and circumvent the tear. The surgical treatment of irreparable RCTs is limited, however, because of the fact that it is irreparable. Without the ability to fully repair and heal the rotator cuff, it is challenging to regain full functionality and strength of the shoulder. However, surgical treatments can provide the opportunity to still have good outcomes that allow for functional use of the shoulder.

The lack of consistency in reported outcome measures poses a challenge in comparing the various treatment methods. The most objective measure reported for comparison of all treatment methods is the success rate of that treatment when performed by capable, skilled surgeons. This review uses the collective failure rates of various treatment methods for irreparable RCTs to compare the outcomes of patients being treated by each method. This review aims to compile failure rates of the different treatment methods and subsequently compare them against each other with the goal of determining if any method is the most successful. This analysis could help further develop an overarching guideline as to which methods should be considered first when weighing the options for treating irreparable RCTs.

## Materials and methods

### Search rationale

The Preferred Reporting Items for Systematic Reviews and Meta-Analyses statement guidelines were meticulously followed in this systematic review.^92^ The MEDLINE, Embase, and Scopus databases were exhaustively searched in July 2024 for studies reporting outcomes of treatment options for irreparable RCTs. The searches were conducted using specific terms to ensure the inclusion of all relevant studies, and the titles, abstracts, and full texts were thoroughly reviewed to identify studies that met the inclusion criteria. The searches included “irreparable rotator cuff tears” along with the treatment methods added for each. For example, “irreparable rotator cuff tears arthroscopic débridement.”

The treatment options were carefully categorized into 7 major treatment groups, including arthroscopic débridement, subacromial balloon spacer, interposition graft repair, partial repair, reverse total shoulder arthroplasty (rTSA), superior capsule reconstruction (SCR), and tendon transfers (including latissimus dorsi and trapezius transfers). Arthroscopic débridement was defined as solely débridement of the RCT with no other repairs made. Subacromial balloon spacer was defined as the insertion of a balloon into the subacromial space. Interposition graft repair was defined as the use of autografts, allografts, xenografts, and synthetic grafts to bridge the tendon in the repair of the irreparable RCT. Partial repair was defined as any attempt made to repair the irreparable RCT, and in the definition of irreparable RCT, all tendons were not able to be completely repaired. rTSA was defined as the insertion of a reverse total shoulder implant. SCR was defined as the repair of the superior capsule, which in most studies included the use of a graft.

### Study eligibility

This review included patients who were treated for large to massive irreparable RCTs. Studies were deemed eligible if they reported outcomes, specifically failure rates, for repairs of large to massive irreparable rotator cuffs. Studies classified as literature reviews, cadaveric studies, case reports, biomechanical studies, editorial commentaries, surgical techniques, animal studies, and studies written in a language other than English were excluded. There were no exclusion criteria based on patient population, and there was no restriction on follow-up periods. In the analysis of bias, the follow-up period was deemed appropriate for every study. Any disagreements were resolved by consensus between the authors.

### Data collection and analysis

Data for each included study were thoroughly reviewed, and the data from the study were collected and compiled into a standardized data collection sheet. For each study, the number of patients, failures, the treatment method, the failure rate, and the treatment for failed patients (when provided) were collected. Failures were defined as retears where appropriate, reoperations about the treatment option, or clinical failures as reported by the studies themselves. In studies that did perform postoperative imaging, the results of the imaging showing retears were classified as failures, which correlated with the results reported in those studies. Not all studies reported postoperative imaging, which resulted in the collection of the failure rate reported based on their individual classification. The data were then separated into their respective treatment methods. Comparisons between the data sets for each treatment option were performed using a chi-square analysis (*P*< .05) (SPSS; IBM, Armonk, NY, USA). Following data collection, the studies were assessed for bias.

### Quality assessment

All included studies were reviewed and assessed by 1 independent reviewer (S.C.) using the Methodological Index for Nonrandomized Studies scoring system (Appendix I).^116^ The studies were ranked in 7 metrics on a scale of 0-2 to evaluate their risk of bias, giving a maximum score of 14. No included studies scored less than an 8, and 86% of studies (117) scored a 10 or higher.

## Results

A total of 136 studies consisting of 4,963 shoulders were included in this review. They were separated into different groups according to the treatment method that was studied. The groups included arthroscopic débridement,^10^^,^^58^^,^^65^^,^^76^^,^^95^^,^^128^ subacromial balloon spacer,^5^^,^^30^^,^^35^^,^^72^, ^73^, ^74^^,^^76^^,^^88^^,^^104^^,^^110^^,^^114^^,^^129^^,^^135^^,^^136^ interpositional graft repair,^3^^,^^4^^,^^14^^,^^22^^,^^43^^,^^44^^,^^56^^,^^81^^,^^82^^,^^86^^,^^88^^,^^93^^,^^107^, ^108^, ^109^^,^^111^^,^^113^^,^^127^ partial repair,^16^^,^^19^^,^^23^^,^^24^^,^^33^^,^^41^^,^^48^^,^^51^^,^^56^^,^^67^^,^^82^^,^^93^^,^^94^^,^^96^^,^^100^^,^^103^^,^^109^^,^^112^^,^^129^ rTSA,^13^^,^^25^^,^^29^^,^^34^^,^^36^^,^^49^^,^^62^^,^^75^^,^^85^^,^^105^^,^^117^^,^^125^^,^^126^^,^^130^ SCR,^1^^,^^8^^,^^15^^,^^31^^,^^32^^,^^41^^,^^47^^,^^57^^,^^59^^,^^61^, ^62^, ^63^, ^64^^,^^66^^,^^68^^,^^70^^,^^77^, ^78^, ^79^, ^80^^,^^89^, ^90^, ^91^^,^^94^^,^^97^^,^^99^^,^^101^^,^^102^^,^^105^^,^^112^^,^^117^^,^^119^^,^^120^ and tendon transfers (includes latissimus dorsi and trapezius transfers).^2^^,^^9^^,^^12^^,^^17^^,^^21^^,^^26^, ^27^, ^28^^,^^37^, ^38^, ^39^^,^^42^^,^^45^^,^^47^^,^^50^^,^^53^, ^54^, ^55^^,^^69^^,^^83^^,^^84^^,^^87^^,^^91^^,^^94^^,^^106^^,^^115^^,^^123^^,^^124^^,^^131^, ^132^, ^133^^,^^137^ Failure rates for each study were collected, and overall failure rates for each treatment were compiled and subsequently analyzed against the other treatment methods.

Table I contains data for each treatment method. The tendon transfer subset included latissimus dorsi transfers (29 studies) and trapezius transfers (3 studies) due to the low number of patients and studies focused on trapezius transfers.

**Table I:** Failure rates of each treatment group.

Treatment method	Included studies	Failures	Total shoulders	Overall failure rate
Arthroscopic débridement	6	24	244	9.8%
Balloon spacer	14	134	396	33.8%
Interpositional graft	18	100	499	20.0%
Partial repair	20	148	705	21.0%
Reverse shoulder arthroplasty	14	103	791	13.0%
Superior capsule reconstruction	32	186	1,303	14.3%
Tendon transfer	32	120	1,025	11.7%

When comparing the failure rates of the treatment groups, it was found that the subacromial balloon spacer had the highest failure rate (*P*< .001). The partial repair group (*P*≤ .001) and the interpositional graft group (*P*≤ .012) had higher failure rates than the arthroscopic débridement, rTSA, SCR, and tendon transfer groups. Still, the partial repair group had a similar failure rate to the interposition graft (*P*= .744). The arthroscopic débridement, rTSA, SCR, and tendon transfer groups reported similar failure rates (*P*≥ .100).

A total of 69 studies reported the subsequent treatments for the patients who suffered failures. The overwhelming majority (84%) of studies that reported subsequent treatment used rTSA for at least some of their patients.

## Discussion

The treatment of irreparable RCTs has recently become a popular literature topic, but there is no current consensus on the best treatment method. Reviews have been conducted to evaluate these treatment methods on patient-reported outcomes, complications, minimal clinically significant differences, and functional outcomes.^7^^,^^20^^,^^52^^,^^60^^,^^71^^,^^118^ Kovacevic et al reported that physical therapy could be inferior to surgical management but could not make a concrete recommendation as to which treatment was superior.^60^

Many studies have compared rTSA, SCR, and tendon transfer, as they are popular for treating irreparable RCTs.^91^^,^^105^ This study's finding of arthroscopic débridement having a failure rate close to these other 3 treatments is new, but it could be misleading. All other groups had well-defined parameters for a retear or failure following surgery. The arthroscopic débridement group, however, had more subjective definitions of failure since no repair or implant can be evaluated as retearing or failing. The individual studies reported subjective clinical failures. This can be misleading, as in the other treatment groups, there were retears of grafts seen on MRIs that had good results but would still be included in the retear count. These patients were included because retears and failures were reported in all of the included studies, but some did not include subjective clinical evaluation of success or failure.

The similar failure rates of rTSA, SCR, and tendon transfers correspond with the varying results in the individual studies that compare between these treatment methods.^47^^,^^91^^,^^105^ It is disappointing that a consensus of what individual treatment is superior could not be made based on failure rate. Still, many factors play into the decision to pursue each treatment option, such as age, activity level, tear characteristics/size, fatty infiltration, arthritis, and the patient's preferences. However, there was some clarity in showing that subacromial balloon spacers, interpositional grafts, and partial repairs have higher failure rates than rTSA, SCR, tendon transfers, and arthroscopic débridement.

This study does have some considerable limitations that prompt the need for future reviews. The inclusion of only failure rates as reported by the studies and not including any clinical outcomes limits the impact of this study, as there are patients who have a mechanical failure of the surgical treatment but still have a clinically good outcome. The opposite could also be true where a patient has an intact repair but does not improve clinically with regaining their shoulder function or decreasing their pain. This choice was reasonably made due to the need for consistency in reported outcomes in order to compare the treatments, but it does provide the opportunity for future focused reviews of individual treatment methods to analyze more outcome measures. Also, not all studies reported postoperative imaging as their classification of a failure allowing for less objective clinical measures to be used in their determination of a failure.

During this review, several studies combined these treatment techniques, such as an rTSA with a latissimus dorsi transfer, to improve the external rotation that the rTSA lacks.^11^^,^^12^ Some studies investigated the outcomes of interposition grafts versus partial repairs or SCR with and without a partial rotator cuff repair incorporated.^82^^,^^134^ There were some promising results seen in these studies. Perhaps rather than determining only one treatment method that yields the most superior results, there may be an opportunity for more research into combining some of these methods to create a technique that yields synergistic results for irreparable RCTs.

## Conclusion

There continues not to be a consistently great answer in ranking which method to use in treating irreparable RCTs. Comparing failure rates in this review showed that rTSA, SCR, tendon transfers, and possibly arthroscopic débridement are the superior options, but failure rates between them are all similar. However, subacromial balloon spacers, interpositional grafts, and partial repairs fail at higher rates than the other group of options. There is a continued need for more research into the effectiveness of treatment methods for irreparable RCTs through analyzing failure rates and functional clinical outcomes.

## Disclaimers:

Funding: No funding was disclosed by the authors.

Conflicts of interest: Jason L. Koh is a shareholder in Marrow Access Technologies, which manufactures a marrow access device sometimes used in rotator cuff repair, which is related to the subject of this article. Any additional authors, their immediate families, and any research foundations with which they are affiliated have not received any financial payments or other benefits from any commercial entity related to the subject of this article.
