# Supplementary material for: An analysis of failure rates for treatment options for large to massive irreparable rotator cuff tears: a systematic review
Source: J Shoulder Elb Arthroplast. 2026 Apr 13;10(1-2):100019. doi: 10.1016/j.jsea.2026.100019 (PMC13157070; doi:10.1016/j.jsea.2026.100019)
Supplement: Supplementary Appendix S1 [file mmc1.docx]

Appendix I: Risk of Bias Evaluation Using MINORS Criteria

Arthroscopic Debridement

| Study | Clearly state aim | Inclusion of consecutive patients | Prospective data collection | Unbiased endpoint assessment | Follow-up appropriate | Loss to f/u | Prospective calculation of sample size |
| --- | --- | --- | --- | --- | --- | --- | --- |
| Boileau^10^ | 2 | 1 | 1 | 2 | 2 | 2 | 0 |
| Klinger^11^ | 2 | 2 | 2 | 2 | 2 | 2 | 0 |
| Lee^12^ | 2 | 2 | 2 | 2 | 2 | 2 | 0 |
| Metcalfe^13^ | 2 | 1 | 2 | 2 | 2 | 2 | 0 |
| Park^14^ | 2 | 1 | 1 | 2 | 2 | 1 | 0 |
| Verhelst^15^ | 2 | 1 | 2 | 2 | 2 | 1 | 0 |

Subacromial Balloon Spacer

| Study | Clearly state aim | Inclusion of consecutive patients | Prospective data collection | Unbiased endpoint assessment | Follow-up appropriate | Loss to f/u | Prospective calculation of sample size |
| --- | --- | --- | --- | --- | --- | --- | --- |
| Metcalfe^13^ | 2 | 1 | 2 | 2 | 2 | 2 | 0 |
| Bakti^16^ | 2 | 1 | 2 | 2 | 2 | 0 | 0 |
| Familiari^17^ | 2 | 1 | 2 | 2 | 2 | 0 | 0 |
| Garriguez-Perez^18^ | 2 | 2 | 0 | 2 | 2 | 2 | 0 |
| Iban^19^ | 2 | 2 | 2 | 2 | 2 | 0 | 0 |
| Malahias^20^ | 2 | 1 | 2 | 2 | 2 | 2 | 0 |
| Malahias^21^ | 2 | 1 | 2 | 2 | 2 | 2 | 0 |
| Maman^22^ | 2 | 0 | 0 | 2 | 2 | 2 | 0 |
| Oh^23^ | 2 | 2 | 2 | 2 | 2 | 2 | 2 |
| Prat^24^ | 2 | 2 | 2 | 2 | 2 | 2 | 0 |
| Senekovic^25^ | 2 | 1 | 2 | 2 | 2 | 1 | 0 |
| Verma^26^ | 2 | 1 | 2 | 2 | 2 | 1 | 0 |
| Yallapragada^27^ | 2 | 1 | 2 | 2 | 2 | 2 | 0 |
| Yamak^28^ | 2 | 0 | 2 | 2 | 2 | 2 | 0 |

Interposition Graft

| Study | Clearly state aim | Inclusion of consecutive patients | Prospective data collection | Unbiased endpoint assessment | Follow-up appropriate | Loss to f/u | Prospective calculation of sample size |
| --- | --- | --- | --- | --- | --- | --- | --- |
| Audenaert^29^ | 2 | 2 | 2 | 2 | 2 | 2 | 0 |
| Badhe^30^ | 2 | 2 | 2 | 2 | 2 | 2 | 0 |
| Bond^31^ | 2 | 2 | 2 | 2 | 2 | 2 | 0 |
| Dukan^32^ | 2 | 2 | 2 | 2 | 2 | 1 | 0 |
| Gupta^33^ | 1 | 2 | 2 | 2 | 2 | 2 | 0 |
| Gupta^34^ | 2 | 2 | 2 | 2 | 2 | 2 | 0 |
| Kawashima^35^ | 2 | 0 | 2 | 2 | 2 | 2 | 0 |
| Modi^36^ | 2 | 2 | 2 | 2 | 2 | 2 | 0 |
| Mori^37^ | 2 | 2 | 2 | 2 | 2 | 2 | 0 |
| Neumann^38^ | 2 | 1 | 2 | 2 | 2 | 1 | 0 |
| Oh^23^ | 2 | 2 | 2 | 2 | 2 | 2 | 2 |
| Pandey^39^ | 2 | 2 | 2 | 2 | 2 | 2 | 0 |
| Rhee^40^ | 2 | 1 | 2 | 2 | 2 | 1 | 0 |
| Rhee^41^ | 1 | 2 | 2 | 2 | 2 | 2 | 0 |
| Ribiero^42^ | 2 | 1 | 2 | 2 | 2 | 2 | 2 |
| Sano^43^ | 2 | 1 | 0 | 2 | 2 | 2 | 0 |
| Seker^44^ | 2 | 1 | 2 | 2 | 2 | 1 | 0 |
| Varvitsiotis^45^ | 2 | 2 | 2 | 2 | 2 | 2 | 0 |

Partial Repair

| Study | Clearly state aim | Inclusion of consecutive patients | Prospective data collection | Unbiased endpoint assessment | Follow-up appropriate | Loss to f/u | Prospective calculation of sample size |
| --- | --- | --- | --- | --- | --- | --- | --- |
| Chen^46^ | 2 | 1 | 1 | 2 | 2 | 1 | 0 |
| Cuff^47^ | 2 | 1 | 1 | 2 | 2 | 1 | 0 |
| Duralde^48^ | 2 | 1 | 2 | 2 | 2 | 2 | 0 |
| Efremov^49^ | 2 | 0 | 2 | 2 | 2 | 1 | 0 |
| Galasso^50^ | 2 | 2 | 2 | 2 | 2 | 0 | 0 |
| Greiner^51^ | 2 | 2 | 2 | 2 | 2 | 2 | 2 |
| Haque^52^ | 2 | 1 | 2 | 2 | 2 | 2 | 0 |
| Jeong^53^ | 2 | 1 | 1 | 2 | 2 | 1 | 0 |
| Kawashima^35^ | 2 | 0 | 2 | 2 | 2 | 2 | 0 |
| Lee^54^ | 2 | 1 | 1 | 2 | 2 | 0 | 0 |
| Malahias^21^ | 2 | 1 | 2 | 2 | 2 | 2 | 0 |
| Mori^37^ | 2 | 2 | 2 | 2 | 2 | 2 | 0 |
| Pandey^39^ | 2 | 2 | 2 | 2 | 2 | 2 | 0 |
| Paribelli^55^ | 2 | 1 | 2 | 2 | 2 | 2 | 0 |
| Park^56^ | 2 | 1 | 2 | 2 | 2 | 2 | 0 |
| Pines^57^ | 2 | 0 | 2 | 2 | 2 | 2 | 0 |
| Porcellini^58^ | 2 | 2 | 2 | 2 | 2 | 1 | 0 |
| Ribiero^42^ | 2 | 1 | 2 | 2 | 2 | 2 | 2 |
| Schanda^59^ | 2 | 1 | 2 | 2 | 2 | 2 | 0 |
| Verma^26^ | 2 | 1 | 2 | 2 | 2 | 1 | 0 |

Reverse Shoulder Arthroplasty

| Study | Clearly state aim | Inclusion of consecutive patients | Prospective data collection | Unbiased endpoint assessment | Follow-up appropriate | Loss to f/u | Prospective calculation of sample size |
| --- | --- | --- | --- | --- | --- | --- | --- |
| Boileau^60^ | 2 | 1 | 2 | 2 | 2 | 2 | 0 |
| Ek^61^ | 2 | 1 | 2 | 2 | 2 | 2 | 0 |
| Ernstbrunner^62^ | 2 | 2 | 2 | 2 | 2 | 1 | 0 |
| Garofalo^63^ | 2 | 1 | 2 | 2 | 2 | 2 | 0 |
| Gerber^64^ | 2 | 2 | 2 | 2 | 2 | 0 | 0 |
| Hartzler^65^ | 2 | 1 | 1 | 2 | 2 | 2 | 0 |
| Lacheta^66^ | 2 | 1 | 2 | 2 | 2 | 2 | 2 |
| Marigi^67^ | 2 | 1 | 2 | 2 | 2 | 0 | 0 |
| Mulieri^68^ | 2 | 1 | 2 | 2 | 2 | 0 | 0 |
| Reddy^69^ | 2 | 1 | 2 | 2 | 2 | 0 | 2 |
| So^70^ | 2 | 1 | 2 | 2 | 2 | 2 | 0 |
| Valenti^71^ | 2 | 1 | 2 | 2 | 2 | 0 | 0 |
| Varvitiotis^72^ | 2 | 1 | 2 | 2 | 2 | 0 | 0 |
| Wall^73^ | 2 | 2 | 2 | 2 | 2 | 2 | 0 |

Superior Capsule Reconstruction

| Study | Clearly state aim | Inclusion of consecutive patients | Prospective data collection | Unbiased endpoint assessment | Follow-up appropriate | Loss to f/u | Prospective calculation of sample size |
| --- | --- | --- | --- | --- | --- | --- | --- |
| Alarcon^74^ | 2 | 2 | 1 | 2 | 2 | 2 | 0 |
| Bi^75^ | 2 | 2 | 2 | 2 | 2 | 2 | 0 |
| Burkhart^76^ | 2 | 2 | 1 | 2 | 2 | 1 | 0 |
| Ferrando^77^ | 2 | 2 | 2 | 2 | 2 | 2 | 0 |
| Gabbott^78^ | 2 | 1 | 2 | 2 | 2 | 2 | 0 |
| Greiner^51^ | 2 | 2 | 2 | 2 | 2 | 2 | 2 |
| Hanson^79^ | 2 | 1 | 2 | 2 | 2 | 1 | 0 |
| Kim^80^ | 2 | 2 | 1 | 2 | 2 | 1 | 0 |
| Kocaoglu^81^ | 2 | 2 | 1 | 2 | 2 | 2 | 0 |
| LaBelle^82^ | 2 | 2 | 1 | 2 | 2 | 1 | 0 |
| Lacheta^66^ | 2 | 1 | 2 | 2 | 2 | 2 | 2 |
| Lacheta^83^ | 2 | 2 | 2 | 2 | 2 | 2 | 0 |
| Ladermann^84^ | 2 | 1 | 2 | 2 | 2 | 2 | 2 |
| Lee^85^ | 2 | 2 | 2 | 2 | 2 | 2 | 0 |
| Lee and Min^86^ | 2 | 2 | 1 | 2 | 2 | 1 | 0 |
| Lim^87^ | 2 | 2 | 2 | 2 | 2 | 2 | 0 |
| Mihata^88^ | 2 | 2 | 2 | 2 | 2 | 2 | 0 |
| Mihata^89^ | 2 | 2 | 2 | 2 | 2 | 2 | 0 |
| Mihata^90^ | 2 | 2 | 2 | 2 | 2 | 0 | 0 |
| Mihata^91^ | 2 | 2 | 2 | 2 | 2 | 2 | 2 |
| Ohta^92^ | 2 | 1 | 1 | 2 | 2 | 1 | 0 |
| Okamura^93^ | 2 | 2 | 2 | 2 | 2 | 2 | 2 |
| Ozturk^94^ | 2 | 2 | 2 | 2 | 2 | 2 | 0 |
| Pashuck^95^ | 2 | 1 | 2 | 2 | 2 | 2 | 0 |
| Pennington^96^ | 2 | 2 | 2 | 2 | 2 | 2 | 0 |
| Polacek^97^ | 2 | 2 | 2 | 2 | 2 | 2 | 0 |
| Polacek^98^ | 2 | 2 | 2 | 2 | 2 | 2 | 0 |
| Reddy^69^ | 2 | 1 | 2 | 2 | 2 | 0 | 2 |
| Schanda^99^ | 2 | 1 | 2 | 2 | 2 | 2 | 0 |
| So^100^ | 2 | 1 | 2 | 2 | 2 | 2 | 2 |
| Takayama^101^ | 2 | 2 | 2 | 2 | 2 | 2 | 0 |
| Takayama^102^ | 2 | 1 | 2 | 2 | 2 | 2 | 0 |

Tendon Transfers

| Study | Clearly state aim | Inclusion of consecutive patients | Prospective data collection | Unbiased endpoint assessment | Follow-up appropriate | Loss to f/u | Prospective calculation of sample size |
| --- | --- | --- | --- | --- | --- | --- | --- |
| Aoki^103^ | 2 | 1 | 2 | 2 | 2 | 2 | 0 |
| Birminghan and Neviaser^104^ | 2 | 2 | 2 | 2 | 2 | 1 | 0 |
| Bolieau^105^ | 2 | 2 | 2 | 2 | 2 | 2 | 0 |
| Chopra^106^ | 2 | 1 | 2 | 2 | 2 | 2 | 0 |
| De Casas^107^ | 2 | 1 | 2 | 2 | 2 | 2 | 0 |
| El-Azab^108^ | 2 | 2 | 2 | 2 | 2 | 0 | 0 |
| Elhassan^109^ | 2 | 1 | 2 | 2 | 2 | 0 | 0 |
| Elhassan^110^ | 2 | 1 | 2 | 2 | 2 | 0 | 0 |
| Gerber^111^ | 2 | 1 | 2 | 2 | 2 | 0 | 0 |
| Gerber^112^ | 2 | 1 | 2 | 2 | 2 | 1 | 0 |
| Gerhardt^113^ | 2 | 2 | 2 | 2 | 2 | 2 | 0 |
| Grimberg^114^ | 2 | 1 | 2 | 2 | 2 | 2 | 0 |
| Habermeyer^115^ | 2 | 2 | 2 | 2 | 2 | 0 | 0 |
| Hanson^79^ | 2 | 1 | 2 | 2 | 2 | 1 | 0 |
| Iannotti^116^ | 2 | 1 | 2 | 2 | 2 | 1 | 0 |
| Kany^117^ | 2 | 1 | 2 | 2 | 2 | 1 | 0 |
| Kany^118^ | 2 | 1 | 2 | 2 | 2 | 1 | 0 |
| Kany^119^ | 2 | 2 | 2 | 2 | 2 | 1 | 2 |
| Lehmann^120^ | 2 | 2 | 2 | 2 | 2 | 2 | 0 |
| Muench^121^ | 2 | 1 | 2 | 2 | 2 | 0 | 2 |
| Moursy^122^ | 2 | 1 | 2 | 2 | 2 | 1 | 0 |
| Nove-josserand^123^ | 2 | 1 | 2 | 2 | 2 | 1 | 0 |
| Ozturk^94^ | 2 | 2 | 2 | 2 | 2 | 2 | 0 |
| Paribelli^55^ | 2 | 1 | 2 | 2 | 2 | 2 | 0 |
| Reinares^124^ | 2 | 1 | 2 | 2 | 2 | 2 | 0 |
| Sidler-Maier^125^ | 2 | 1 | 2 | 2 | 2 | 2 | 0 |
| Valenti^126^ | 2 | 2 | 2 | 2 | 2 | 2 | 0 |
| Valenti^127^ | 2 | 2 | 2 | 2 | 2 | 2 | 0 |
| Waltenspul^128^ | 2 | 2 | 2 | 2 | 2 | 1 | 0 |
| Warner^129^ | 2 | 1 | 2 | 2 | 2 | 0 | 0 |
| Weening and Willems^130^ | 2 | 1 | 2 | 2 | 2 | 1 | 0 |
| Zafra^131^ | 2 | 1 | 2 | 2 | 2 | 2 | 0 |
